# Structural and Functional Insights into the Roles of Potential Metal-Binding Sites in *Apostichopus japonicus* Ferritin

**DOI:** 10.3390/polym14245378

**Published:** 2022-12-08

**Authors:** Yan Wu, Chunheng Huo, Tinghong Ming, Yan Liu, Chang Su, Xiaoting Qiu, Chenyang Lu, Jun Zhou, Ye Li, Zhen Zhang, Jiaojiao Han, Ying Feng, Xiurong Su

**Affiliations:** 1 State Key Laboratory for Managing Biotic and Chemical Threats to the Quality and Safety of Agro-Products, Ningbo University, Ningbo 315211, China; 2 College of Food and Pharmaceutical Sciences, Ningbo University, Ningbo 315832, China; 3 Key Laboratory of Aquacultural Biotechnology Ministry of Education, Ningbo University, Ningbo 315832, China; 4 School of Marine Science, Ningbo University, Ningbo 315832, China; 5 Zhejiang Collaborative Innovation Center for High Value Utilization of Byproducts from Ethylene Project, Ningbo Polytechnic, Ningbo 315800, China; 6 College of Life Sciences, Tonghua Normal University, Tonghua 134000, China

**Keywords:** *Apostichopus japonicus* ferritin, potential metal-binding sites, mutant, small-angle X-ray scattering, crystallography, catalytic activity

## Abstract

Ferritin is widely acknowledged as a conservative iron storage protein found in almost all living kingdoms. *Apostichopus japonicus* (Selenka) is among the oldest echinoderm fauna and has unique regenerative potential, but the catalytic mechanism of iron oxidation in *A. japonicus* ferritin (AjFER) remains elusive. We previously identified several potential metal-binding sites at the ferroxidase center, the three- and four-fold channels in AjFER. Herein, we prepared AjFER, AjFER-E25A/E60A/E105A, AjFER-D129A/E132A, and AjFER-E168A mutants, investigated their structures, and functionally characterized these ferritins with respect to Fe^2+^ uptake using X-ray techniques together with biochemical analytical methods. A crystallographic model of the AjFER-D129A/E132A mutant, which was solved to a resolution of 1.98 Å, suggested that the substitutions had a significant influence on the quaternary structure of the three-fold channel compared to that of AjFER. The structures of these ferritins in solution were determined based on the molecular envelopes of AjFER and its variants by small-angle X-ray scattering, and the structures were almost consistent with the characteristics of well-folded and globular-shaped proteins. Comparative biochemical analyses indicated that site-directed mutagenesis of metal-binding sites in AjFER presented relatively low rates of iron oxidation and thermostability, as well as weak iron-binding affinity, suggesting that these potential metal-binding sites play critical roles in the catalytic activity of ferritin. These findings provide profound insight into the structure–function relationships related to marine invertebrate ferritins.

## 1. Introduction

Iron is considered to be one of the essential trace elements necessary for all living organisms and it is involved in many indispensable functions in many life forms, such as electron transfer, oxygen transport, DNA synthesis, and detoxication reaction [1]. Although it is necessary and involved in these functions, iron is potentially toxic in the body when present in excessive amounts owing to its low solubility in the stable oxidation state (i.e., Fe^3+^) and its tendency to potentiate the production of high levels of oxidative stress [2,3,4]. Accordingly, it is crucially important to maintain a dynamic balance between the benefits and toxic effects of iron by tightly regulating iron homeostasis, which is mostly accomplished by iron-binding proteins such as ferritin and transferrin [3,5].

Ferritins are well known as an ancient superfamily of globular proteins that are associated with the storing and detoxification of iron. In addition, they exhibit a capability to oxidize Fe^2+^ to generate Fe^3+^; and ferritins facilitate iron nucleation as their mineral core for storage inside the protein cavity, allowing the accommodation of up to 4500 iron atoms [5]. In general, ferritin’s high capacity for iron storage is mainly due to antioxidant protection, which is achieved by preventing Fenton reactions and thus stopping the formation of the radical element hydroxyl (OH⸱), and in the reversible storage of iron [3,4]. Notably, the superfamily of these proteins is usually classified into three different types, i.e., classical ferritins, heme-binding bacterioferritins (Bfr), and DNA-binding proteins from starved cells (Dps) [6]. The typical form of most common ferritins presents as a spherical protein composed of 24 identical or similar subunits with a molecular weight (MW) of ~450−500 kDa [7]. In eukaryotes, ferritin is generally formed by self-assembly with two or three types of highly homologous subunits, namely heavy (H, 21 kDa), middle (M, 20 kDa), and light (L, 19 kDa) chains, in which the M subunit contains both the ferroxidase center and the ferrihydrite nucleation center, in stark contrast to the H and L subunits [4,6]. Specifically, M-ferritins are found in some lower vertebrate (e.g., amphibians and fish) and invertebrate (e.g., shellfish) species and closely resemble vertebrate H-ferritins (sequence identity of ~85%) [6,8]. Previous research has indicated that the marine invertebrate *Apostichopus japonicus* ferritin (AjFER) shows very high sequence similarity with other invertebrates and comprises known conserved functional domains of both the di-iron ferroxidase sites of the H subunit and the iron nucleation site of the L subunit [8,9,10], suggesting that AjFER is functionally similar to M-ferritin. However, knowledge regarding the iron uptake and oxidation mechanism of M-ferritins from marine invertebrates remains limited.

Structurally, each subunit of almost all ferritins is composed of a characteristic anti-parallel *α*-helix bundle (helices A–D) and a fifth short E-helix (with the exception of Dps proteins) pointing inside the protein cavity [11]. These subunits assemble into a spherical structure with octahedral (4-3-2) symmetry, resulting in 12 two-fold, 8 three-fold, and 6 four-fold channels. Specifically, the hydrophobic three-fold channel connecting the inner cavity to the outside in ferritins is considered a potential gateway for the entry and exit of iron ions and other small molecules [5,11,12]. Numerous studies have demonstrated that the carboxylate side chains of Asp and Glu residues along the three-fold pores are essential for iron uptake, and these residues are involved in the translocation of Fe^2+^ ions toward the ferroxidase center for rapid catalytic activity in human H-ferritin (HuHF) and frog M-ferritin [6,12]. Moreover, a previous X-ray crystal structure also indicated that the Asp129 and Glu132 residues of the three-fold channel in AjFER, corresponding to that of highly conserved residues in other marine invertebrates [13,14], indeed play a key role in iron uptake [9]. Of course, after uptake, the Fe^2+^ ions are transported by a series of metal-binding residues toward a specific catalytic site, which is known as the di-iron ferroxidase center and is situated in the subunit four-helix bundle [5,9,13,14,15]. However, although the amino acid residues located at the ferroxidase center seem to be highly conserved in most eukaryotic ferritins [7,9], the ferroxidase reaction rate may vary. For instance, De Meulenaere et al. found that the ferroxidase velocity of the marine invertebrate *Chaetopterus* ferritin (ChF) was up to eight times faster than that of HuHF, which is known as a conventionally accepted representative ferritin [15]. Furthermore, recent studies have suggested that the Glu168 residue at the four-fold channel can act as a potential binding site of metal ions in some marine invertebrate ferritins [9,14,15], but there is still a lack of sufficient evidence to determine whether this metal-binding site is related to the catalytic function of ferritin. Accordingly, some unique functions of marine invertebrate ferritins have not been observed.

In this study, on the basis of the previously identified potential metal-binding sites of AjFER, we created the AjFER-E25A/E60A/E105A mutant with three amino acid substitutions at the ferroxidase center, the AjFER-D129A/E132A mutant with two amino acid substitutions at the three-fold channel, and the AjFER-E168A mutant with a single amino acid substitution at the four-fold channel using site-directed mutagenesis. We then determined the crystal structure of the AjFER-D129A/E132A mutant using X-ray crystallography. We further employed small-angle X-ray scattering (SAXS), which is one of the most powerful techniques, to obtain the structures of these proteins in solution. In addition, we performed biochemical characterizations related to the Fe^2+^ oxidation kinetics and metal ion-binding thermodynamics in comparison with AjFER. The present investigation aimed to provide new insight into the roles of potential metal-binding sites in AjFER.

## 2. Materials and Methods

### 2.1. Protein Expression and Purification

The expression construct for AjFER (pET-28a-AjFER) was produced in a previous work [9]. Based on the crystal structure of AjFER, the pET-28a plasmid carrying the AjFER gene was used to introduce the AjFER-E25A/E60A/E105A mutant (MF), AjFER-D129A/E132A mutant (M3), and AjFER-E168A mutant (M4) using gene synthesis. All constructs were verified by gene sequencing. For overproduction of the AjFER variants, the constructs were transformed into *Escherichia coli* BL21(DE3) cells (Thermo Fisher Scientific, Waltham, MA, USA).

The cells were grown at 37 °C in Luria–Bertani medium supplemented with 30 μg/mL kanamycin and 34 μg/mL chloromycetin until the OD_600_ reached 0.6–0.8. Protein expression was initiated with the addition of isopropyl-β-D-thiogalactopyranoside (IPTG, 0.5 mM final concentration) and the cells were then incubated at 18 °C for 20 h. The cells were harvested by centrifugation at 6500× *g* for 20 min at 4 °C and resuspended in 25 mM Tris, pH 8.0, containing 150 mM NaCl and 0.5% (*v/v*) Triton X-100. The cells were disrupted by sonication, and debris was removed by centrifugation at 12,000× *g* for 20 min at 4 °C. Contaminating proteins were removed using a Ni–NTA affinity column (GE Healthcare, Fairfield, CT, USA), and the His-SUMO tag was cleaved by incubation overnight at 4 °C with SUMO protease. The sample purity and oligomerization state were assessed using 12% sodium dodecyl sulfate–polyacrylamide gel electrophoresis (SDS–PAGE). The purified proteins were then concentrated using a 30 kDa molecular weight cutoff Amicon Ultra filter unit (Merck Millipore, Billerica, MA, USA). The protein concentrations were determined using a BCA assay kit (Beyotime, Shanghai, China) with bovine serum albumin as the standard.

### 2.2. Circular Dichroism (CD) Spectroscopy

The protein concentrations were adjusted to 0.1 mg/mL with binding buffer (25 mM Tris–HCl, pH 8.0, containing 150 mM NaCl). The CD spectra of AjFER and its variants were examined using a Jasco J-1500 CD spectrometer (Jasco, Tokyo, Japan) with a 0.1 cm path length at room temperature. The far-UV region was scanned from 190 to 260 nm in triplicate with a bandwidth of 1 nm. The CD spectral data are presented as the molar ellipticity (deg∙cm^2^∙dmol^−1^) versus wavelength [θ]. The content of secondary structure elements, including *α*-helixes, *β*-sheets, *β*-turns, and random coils, was analyzed using Yang’s method by Jasco-Corp.

### 2.3. Dynamic Light Scattering (DLS) Experiments

The DLS data were collected on a Zetasizer Nano Zs instrument (Malvern Instruments Ltd., Malvern, UK) using disposable polystyrene microcuvettes (VWR). The protein samples (1 mg/mL) in 25 mM Tris–HCl, pH 8.0, containing 150 mM NaCl were centrifuged at 12,000× *g* for 10 min at room temperature and then loaded into a quartz cuvette prior to measurement. The hydrodynamic diameter (D_H_) distribution of the prepared samples was calculated using Omni SIZE 2.0 software. Three measurements were performed with the instrument optimizing the number of runs for each measurement.

### 2.4. Transmission Electron Microscopy (TEM)

The purified recombinant AjFER and its variants were placed on carbon-coated copper grids. After removing the excess solution by blotting with filter paper, the samples were negatively stained with 1% uranyl acetate for 1 min. The TEM images were captured at 80 kV using a Model H-7650 transmission electron microscope (Hitachi, Tokyo, Japan).

### 2.5. Crystallization, Data Collection and Structure Determination

The preliminary screening for the crystallization conditions was optimized using Crystal Screen I and II (Hampton Research, Riverside, CA, USA) with the sitting-drop vapor-diffusion method. A volume of 1 μL protein solution (approx. 15 mg/mL in 25 mM Tris-HCl, pH 8.0, containing 150 mM NaCl) was mixed with 1 μL reservoir solution in the well of a 48-well plate and incubated at 18 °C. The M3 protein crystals were obtained in a solution containing 0.05 M cadmium sulfate hydrate, 0.1 M HEPES, pH 7.5, and 1.0 M sodium acetate trihydrate. These crystals were harvested using a CryoLoop (Hampton Research). After soaking in a cryoprotection solution supplemented with 20% (*v/v*) glycerol, the crystals were immediately flash-cooled and stored in liquid nitrogen. X-ray data were collected on a BL19U1 beamline with a Pilatus3 6M detector at SSRF [16]. The X-ray wavelength was set to 0.97892 Å. The diffracting data were indexed, integrated, and scaled using the HKL-3000 program suite [17]. A model of the structure was automatically generated by molecular replacement using the BABLES server [18]. The manual model building was performed using COOT software [19], and the automated model building and refinement were conducted using the REFMAC5 program in the CCP4 suite [20]. The superimposed structure of the ferritins was determined using the superpose online server (http://superpose.wishartlab.com/, accessed on 27 June 2022) [21]. All crystallographic figures were generated using the PyMOL molecular graphics system [22]. The collected data and final refinement statistics are summarized in Table 1.

### 2.6. Small-Angle X-ray Scattering (SAXS) Analysis

To study the conformation of ferritins in solution, SAXS experiments were performed at the BL19U2 beamline of the National Center for Protein Science Shanghai (NCPSS) at the Shanghai Synchrotron Radiation Facility (SSRF). The SAXS data were collected as 20 × 1 s exposures, and the scattering profiles for the 20 passes were compared at 10 °C using 60 μL sample in 25 mM Tris-HCl, pH 8.0, containing 150 mM NaCl. A total of 1500 successive frames were recorded using a Pilatus 1 M detector (Dectris Ltd., Baden, Switzerland) with an exposure time of 1.0 s for each frame. The wavelength of the incoming monochromatic X-ray radiation was 1.24 Å with a *q*-range from 0.0084 to 0.4764 Å^−1^ (*q* = 4π⸳sinθ/λ, where 2θ is the scattering angle). All measurements were performed at 293 K. Protein samples with different concentrations (1, 2, 2.5, and 4 mg/mL) in buffer (25 mM Tris–HCl, pH 8.0, containing 150 mM NaCl) were prepared for the X-ray scattering analysis. SAXS data of the protein samples were collected between each buffer. The 2D scattering images were converted to 1D SAXS curves using the BioXTAS RAW software package [23]. The scattering data were processed for background subtraction, concentration scaling, and curve merging using the PRIMUS program in the ATSAS software package [24]. The scattering intensity extrapolated to zero angle *I*(0), pair distance distribution function *p(r)*, radius of gyration *R_g_*, and maximum dimension *D_max_* were determined by Guinier analysis and primus distance distribution analysis using GNOM in ATSAS [25,26]. The data were converted into Kratky plots [*I*⁎*q*^2^ vs. *q*] to evaluate the shape and fold of the proteins, providing information regarding the oligomeric state of the biomolecule [27]. The molecular weights were calculated using the SAXSMow2 program [28]. Ten low-resolution ab initio shape models were calculated using the DAMMIF program [29]. The bead models were generated using the DAMMIN program [30] and the MultiFoXS server [27]. The docking of the sample crystal structures into the SAXS envelopes was superimposed using the SUPCOMB program and visualized using PyMOL [22]. The structural parameters and processed SAXS data are summarized in Table S3.

### 2.7. Iron Oxidation Assay

The iron oxidation assay for AjFER and its variants was conducted by measuring an increase in ultraviolet (UV) absorbance at 315 nm with a UV–visible (UV–vis) spectrophotometer (Shanghai Mapada Instruments Co., Ltd., Shanghai, China) [31]. Oxygen-free aliquots of Fe^2+^ ion (7.2 mM) samples were prepared by dissolving FeSO_4_·7H_2_O in 0.1% (*v/v*) HCl under anaerobic conditions. The protein samples were diluted to a final concentration of 0.5 μM in 20 mM HEPES, pH 7.0, containing 150 mM NaCl. The ferritins and Fe^2+^ ions were added to a 1 mL quartz cuvette (Hellma) at final concentrations of 0.357 and 205.7 μM, respectively. The Fe/protein molar ratio was 576. All experiments were repeated in triplicate.

### 2.8. Inductively Coupled Plasma–Mass Spectrometry (ICP–MS) and CD Analyses

The metal ion content of the protein samples was then quantified with three replicates using a Thermo X Series II ICP–MS instrument (Thermo Fisher Scientific Inc., MA, USA). The standard curves for the Fe atoms were obtained using multielement standard solutions. The atoms per ferritin cage were calculated as previously described [32]. Then, the samples were subjected to CD spectroscopy for secondary structure analysis, as described above.

### 2.9. Microscale Thermophoresis (MST) Measurements

MST, a powerful technique for characterizing biomolecular interactions, depends on the thermophoresis principle of detecting optical fluorescence properties to quantify the binding affinity of different molecules [33]. To determine the binding affinity of Fe^2+^ ions toward AjFER and its variants, MST experiments were performed using a Monolith NT.115 (NanoTemper Technologies, Munich, Germany) and standard capillaries. Each protein sample was labeled using a Protein Labeling Kit RED-NHS 2nd Generation (NanoTemper Technologies, Munich, Germany; catalog no. MO-L011) according to the manufacturer’s instructions. Briefly, 90 µL of protein solution (10 µM) in 20 mM HEPES, pH 7.0, was mixed with 10 µL of 300 µM RED-NHS fluorophore in labeling buffer and incubated for 30 min in the dark at room temperature. Then, the labeled protein was centrifuged at 12,000× *g* at 4 °C for 10 min to remove the protein aggregates. Five microliters of the labeled protein (a concentration of approx. 200 nM) and increasing concentrations of non-labeled ferrous solution (from 3.05 nM to 10 µM) were loaded into NT.115 standard treated capillaries (NanoTemper Technologies, Munich, Germany; catalog no. MO-K022). Subsequently, the capillaries were inserted into the chip tray of the MST instrument, followed by thermophoresis analysis and the appraisal of the binding affinity (K_d_) values. The MST measurements were performed in triplicate at 25 °C, with medium MST power and the red channel using 20% excitation power. Normalization of the fluorescence signal and curve fitting were carried out using MO Affinity Analysis v2.3 software (NanoTemper Technologies, Munich, Germany).

### 2.10. Thermal Stability Analysis

The thermostability of the AjFER, MF, M3, and M4 proteins was determined using a label-free thermal shift assay with the Tycho NT.6 (NanoTemper Technologies, Munich, Germany) via the intrinsic tryptophan and tyrosine fluorescence. The 10 µL solutions of protein samples (approx. 5 mg/mL) were prepared and loaded into Tycho NT.6 capillaries (NanoTemper Technologies, Munich, Germany; catalog no. TYC001). The thermal profiles of the proteins were recorded during a quick thermal ramp from 35 to 95 °C, with a heating rate of 30 °C/min. The inflection unfolding temperatures (T_i_) were assessed based on the changes in the 350/330 nm fluorescence emission ratio values. Thereafter, AjFER and its variants were heated at 90 °C for 10 min, and their structural integrity was analyzed using TEM and DLS methods, as described above.

### 2.11. Statistical Analysis

All data were plotted using GraphPad 8.3 (San Diego, CA, USA) and OriginPro 9.0 (Northampton, MA, USA) software. Significant differences were determined by one-way ANOVA. All data were collected in triplicate and are presented as the mean ± standard deviation (SD). A *p*-value of less than 0.05, 0.01, and 0.001 was considered statistically significant.

## 3. Results

### 3.1. Preparation and Characterization of AjFER and Its Variants

**Figure 1:**
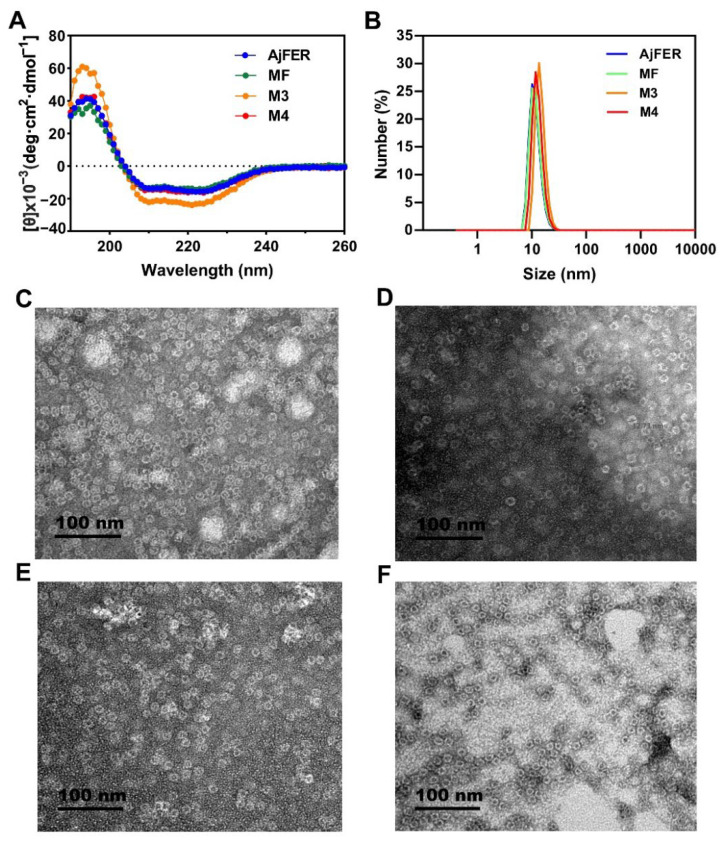
Characterization of the AjFER, MF, M3, and M4 proteins: (**A**) CD spectra of the AjFER, MF, M3, and M4 proteins; (**B**) DLS analyses of the AjFER, MF, M3, and M4 proteins; (**C**–**F**) TEM images of the AjFER, MF, M3, and M4 proteins.

Based on the known structure of AjFER [9], the MF, M3, and M4 proteins were constructed because highly conserved amino acid residues in AjFER were implicated as potential metal-binding sites [9], in which the residues Glu25, Glu60, and Glu105 originated from the ferroxidase center, the residues Asp129 and Glu132 were located inside the three-fold channel, and the Glu168 residue lay at the four-fold channel. The recombinant proteins were characterized by SDS–PAGE analysis for purity verification, CD analysis for secondary conformation determination, DLS analysis for nanoparticle size measurement, and TEM analysis for structural integrity (Figure 1). The SDS–PAGE results indicated that all three variants were composed of each subunit with a molecular weight of approx. 20 kDa (Figure S1). The CD spectra of AjFER and its variants revealed a positive band near 205 nm and two negative bands at approximately 208 and 222 nm, as shown in Figure 1A, suggesting similar secondary structures. The *α*-helix, *β*-sheet, *β*-turn, and random coil contents were calculated, as shown in Table S1. The *α*-helix content decreased/increased non-significantly after mutation (*p* > 0.05). However, the MF protein displayed a decrease of 4.1% for the *β*-turn content and an increase of 3.1% for the *β*-sheet content and 2.8% for the random coil content, compared to AjFER. As a next step, the quaternary structure of the proteins was analyzed in solution at pH 8.0 by DLS analysis (Figure 1B). The results showed that the D_H_ values determined for the MF, M3, and M4 proteins were 11.8 ± 2.8, 14.4 ± 3.0, and 12.9 ± 2.9 nm, respectively, suggesting that the sizes of the variant ferritins were comparable to that of AjFER (11.6 ± 2.8 nm). Subsequently, TEM was used to determine the inherent shell-like structural integrity of the proteins, as depicted in Figure 1C–F. The TEM images of the negatively stained ferritins confirmed that AjFER and its variants presented shell-like structures.

### 3.2. Crystal Structure of the AjFER-D129A/E132A Mutant

Based on the previously reported crystal structure of AjFER [9], the residues Asp129 and Glu132 could be capable of forming electrostatic gradient rings to strongly capture and direct Fe^2+^ ions from the outside environment into the cage; however, the limited crystal data were insufficient to elucidate the underlying ion transport mechanism. On this basis, we expressed and purified the M3 protein, successfully obtained the crystal of the M3 protein, and solved the three-dimensional structure at a resolution of 1.98 Å. The diffraction dataset of the M3 protein was integrated into the monoclinic space Group *C*121, with unit cell parameters of *a* = 192.83 Å, *b* = 130.95 Å, *c* = 122.62 Å, *α* = 90.00°, *γ* = 90.00°, and *β* = 119.40° and with twelve monomers in the asymmetric unit. The crystallographic parameters for the collected diffraction data and structure refinement are summarized in Table 1.

**Figure 2:**
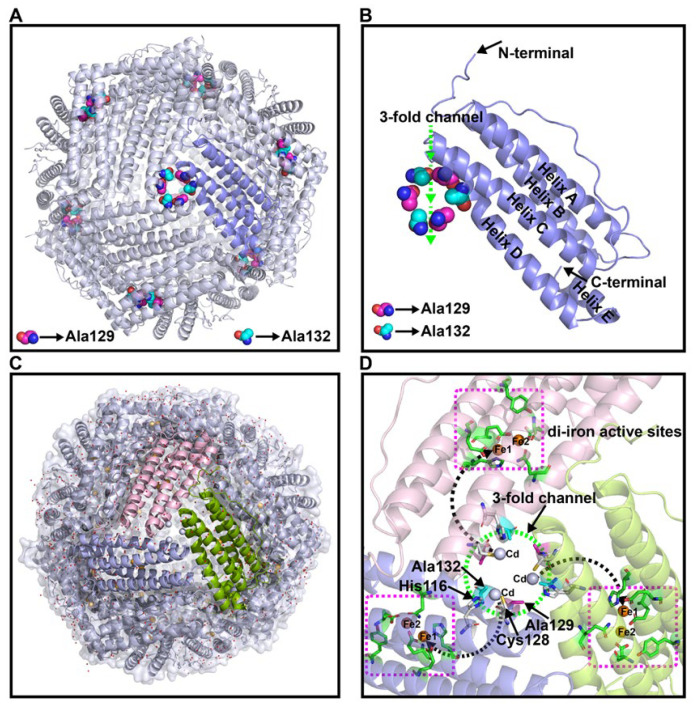
The crystal structure of the M3 protein: (**A**) overall structure view from the outside of the three-fold channel; (**B**) a single subunit of the M3 protein viewed from a ring composed of the D129A/E132A substitutions in the three-fold channel; (**C**) stereo view of the M3 protein nanocage; (**D**) schematic overview of incoming metal ions moving via the three-fold channel from outside the M3 protein cage. The arrows indicate the connections from the three-fold channel toward ferroxidase sites in which yellow and light-blue spheres represent iron and cadmium ions, respectively.

Similar to the assembly of AjFER (PDB ID: 7VHR) [9], the macromolecular M3 protein exhibited a cage-like hollow shell that was composed of 24 subunits and was related by 4-3-2 symmetry (Figure 2A). As previously reported by Behera et al. [34], although the residues Ala129 and Ala132 that stem from the D-helix in the M3 protein subunit generate larger diameters for the three-fold ion channels to facilitate Fe^2+^ movement, they also prevent the guiding of carboxylate side chains that seemingly dominates Fe^2+^ ion traffic (Figure 2B). Only one iron ion was identified in the ferroxidase center in AjFER, most likely because of the relatively low resolution [9], whereas two sites for metal ion binding were observed in the crystal structure of the M3 protein (i.e., sites A and B of the ferroxidase center) (Figure 2C,D). Furthermore, the structural data also indicated the presence of three Cd^2+^ ions inside the three-fold pore, which originated from the crystallization buffer and were mainly coordinated by the side chains of the three symmetry-related His116 and Cys128 residues (Figure 2D). 

**Figure 3:**
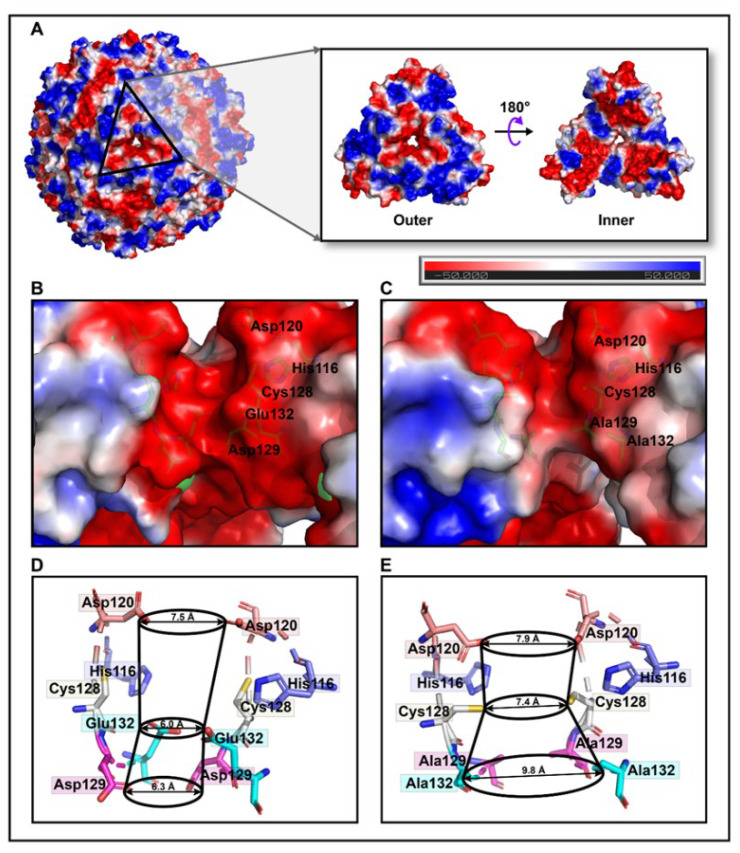
Electrostatic surface potential and nanoscale analyses along the three-fold channel of the M3 protein. (**A**) Overall view of the electrostatic surface potential from outside to inside the cage in the three-fold channel. Comparison of the electrostatic surface potential distribution views at the whole three-fold channel in (**B**) AjFER and (**C**) M3 proteins. The potential scale is rendered from −50 to +50 kTe^−1^ from red to blue. A comparative analysis of the diameter at the three-fold channel in the (**D**) AjFER and (**E**) M3 proteins.

Conventionally, it is a widespread belief that electrostatic gradients are of crucial importance for the overall functioning of proteins [35]. The molecular surface of the M3 protein forming a large hole along the three-fold channel showed dominantly negative potentials lining from the outer to the interior surface (Figure 3A). Rather than the shape of cylindrical channel-like architectures, the three-fold ion channels of the AjFER and M3 proteins were constricted in the middle, similar to an hourglass (Figure 3B,C). Obviously, the inner entrance of the three-fold channel (opening into the hollow cavity) for the M3 protein exhibited relatively neutral electrostatic potential in stark contrast to that of the predominantly negative regions in AjFER. Further results indicated that the three-fold channels of both the AjFER and M3 proteins consisted of three three-fold amino acid rings. The former was composed of three residues: Asp120 (diameter of ~7.5 Å), Glu132 (diameter of ~6.0 Å), and Asp129 (diameter of ~6.3 Å), whereas the latter varied significantly and was composed of three residues Asp120 (diameter of ~7.9 Å), Cys128 (diameter of ~7.4 Å), and Ala129/Ala132 (diameter of ~9.8 Å) based on the calculation results (Figure 3D,E).

**Figure 4:**
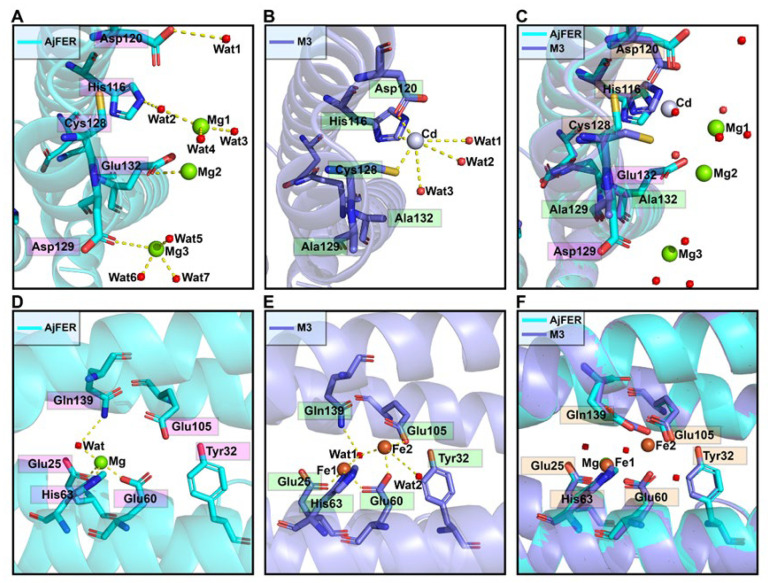
Stereo view and structural superposition of the metal ion coordination environment at the ferroxidase center and the three-fold channel in the AjFER and M3 proteins. Metal ion coordination environment at the three-fold channel of the (**A**) AjFER (PDB code: 7VHR) and (**B**) M3 (PDB code: 7Y74) proteins. (**C**) Superposition of the metal ion coordination environment of the three-fold channel between the AjFER and M3 proteins. The green and light blue spheres represent the magnesium and cadmium ions, respectively, while the red spheres represent water molecules. The metal ion coordination environment at the ferroxidase center in the (**D**) AjFER and (**E**) M3 proteins. (**F**) Superposition of the metal ion coordination environment of the ferroxidase center between the AjFER and M3 proteins. Mg^2+^, Cd^2+^ and Fe^2+^ ions are indicated by green, light blue, and orange spheres, respectively, while the water molecules are represented by red spheres.

The refined structure revealed that the three Cd^2+^ ions were bound to the three-fold channel of the M3 protein nanocage by metal coordination bonds, in contrast to the coordination by three Mg^2+^ ions in AjFER (Figure 4A,B). The binding sites were located on the D-helix at the three-fold axis channels of the M3 protein, at which the Cd^2+^ ion was coordinated by residues His116, Asp120, and Cys128 at distances of 2.33 ± 0.06 Å, 2.47 ± 0.21 Å, and 2.37 ± 0.06 Å from all three symmetrical subunits, respectively, and by three water molecules (Cd–Wat1 distance of 3.70 ± 0.10 Å, Cd–Wat2 distance of 3.53 ± 0.15 Å, and Cd–Wat3 distance of 3.50 ± 0.10 Å) (Figure 4B and Table S2). Structural superimposition for the AjFER and M3 proteins led to a root mean square deviation (RMSD) value of 0.21 Å, illustrating that the location information of the amino acid residues, apart from the Cys128 residue from the three-fold symmetrical subunits, remained relatively stable before and after site-directed mutation (Figure 4C). Notably, the resulting M3 protein structure indicated that two Fe^2+^ ions, which were distinct from AjFER, were involved in coordination with the residues at the ferroxidase center (Figure 4D–F). As illustrated in Figure 4E, Fe1 was bound to the ferroxidase site in coordination with residues Glu25 (distance of 2.17 ± 0.15 Å), Glu60 (distance of 2.23 ± 0.06 Å), His63 (distance of 2.30 ± 0.10 Å), and one water molecule (Fe1–Wat1 distance of 2.60 ± 0.10 Å) (Table S2). Fe2 was coordinated by residues Glu60 (distance of 2.30 ± 0.10 Å), Glu105 (distance of 2.37 ± 0.06 Å), and two water molecules (Fe2–Wat1 distance of 2.37 ± 0.06 Å and Fe2–Wat2 distance of 3.57 ± 0.15 Å).

### 3.3. Structures of AjFER and Its Variants in Solution

**Figure 5:**
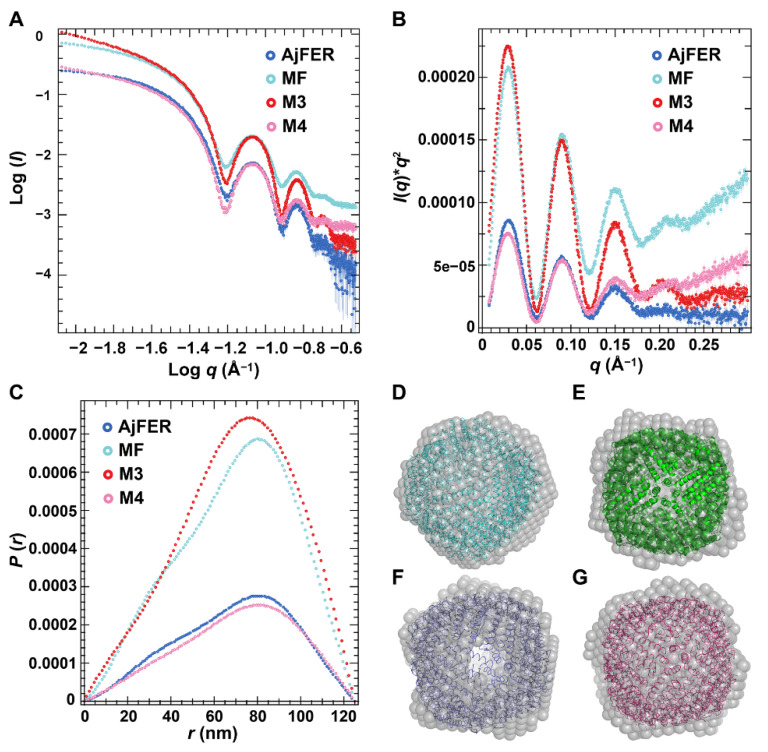
SAXS analysis of AjFER and its variants: (**A**) experimental SAXS scattering curves of the protein samples; (**B**) Kratky plot analysis; (**C**) pair distance distribution curves of the protein samples. Overlay of the filtered and averaged (gray) envelopes (calculated from the SAXS data) of the (**D**) AjFER, (**E**) MF, (**F**) M3, and (**G**) M4 proteins with the crystal structure of AjFER (PDB code: 7VHR).

SAXS analysis was performed to reveal the conformation of AjFER and its variants in solution. The scattering profiles of the AjFER, MF, M3, and M4 proteins presented similar oscillation peaks (Figure 5A), which was consistent with a nanoparticle shaped as a hollow sphere, indicating that they were properly folded and homogeneous globular proteins in solution [36]. Based on the scattering data (Figure 5A–C), the MWs of the AjFER, MF, M3, and M4 proteins were estimated to be 481.3, 510.2, 882.2, and 602.8 kDa, respectively (Table S3). The Kratky plot analysis of the AjFER, MF, M3, and M4 proteins showed three major symmetrical peaks, *q* < 0.18 Å^−1^, which did not decay to near zero at higher *q*-values and maintained a slight elevation, as expected for hollow spheres (Figure 5B). The pair distance distribution function *p(r)* can provide the radius of gyration (*R_g_*), which is based on the full scattering curve and gives the *D_max_* value of a protein as the distance where *p(r)* approaches zero (Figure 5C). The *D*_max_ values from the *p(r)* of the AjFER, MF, M3, and M4 proteins were 120, 121, 125, and 126 Å, respectively (Figure 5C and Table S3), which were typical of hollow spheres supplemented with a maximum shift toward a distance larger than *D_max_*/2 [37]. The radius of gyration values estimated from the Guinier analysis varied between 52.87 and 53.81 Å, which was consistent with that derived from the *p(r)* calculation by GNOM (ranging from 52.61 to 53.54 Å) (Table S3). When fitting the crystal structure of AjFER (PDB code: 7VHR) [9] into the SAXS envelopes of the AjFER, MF, M3, and M4 proteins, similar overall shapes and dimensions were revealed in total (Figure 5D–G).

### 3.4. Biochemical Properties of AjFER and Its Variants

**Figure 6:**
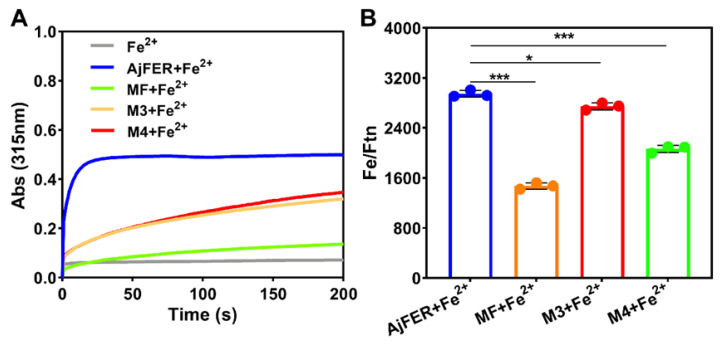
Iron uptake analysis of AjFER and its variants. (**A**) Iron oxidation curves of the protein samples. The oxidation of Fe^2+^ to Fe^3+^ ions was monitored by an increase in the absorbance at 315 nm. (**B**) Determination of iron content among protein samples: Fe^2+^-loaded AjFER (AjFER+Fe^2+^), Fe^2+^-loaded MF protein (MF+Fe^2+^), Fe^2+^-loaded M3 protein (M3+Fe^2+^), and Fe^2+^-loaded M4 protein (M4+Fe^2+^). The molar ratios of iron ions vs. ferritin (Fe/Ftn) were calculated by ICP–MS and the ferritin content per unit volume was determined using a BCA kit. Significant differences are indicated by * (*p* < 0.05), or *** (*p* < 0.001).

**Figure 7:**
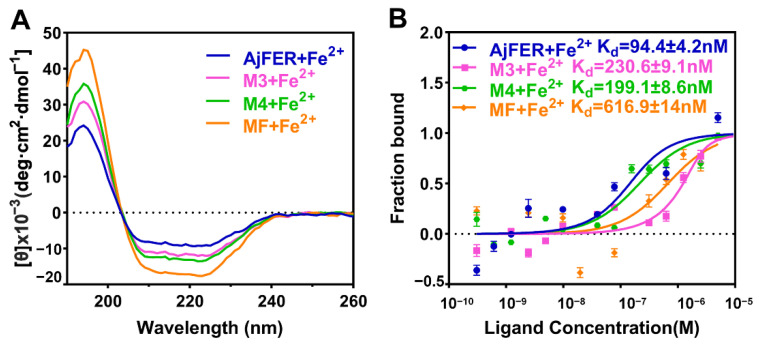
Interactions of AjFER and its variants with Fe^2+^ ions analyzed by (**A**) CD and (**B**) MST. For MST analysis, the error bars represent the mean ± SD in accordance with three independent measurements. Both binding curves and K_d_ values are shown.

The oxidation capacity of ferrous iron by the recombinant proteins was monitored by observing the increase in the absorbance at 315 nm after incubating the proteins with Fe^2+^ ions, as shown in Figure 6A. Compared with AjFER, both the M3 and M4 proteins were capable of oxidizing ferrous iron, but the MF protein almost lost the capacity to oxidize Fe^2+^ ions. Notably, the oxidized activity of the M3 and M4 proteins decreased by more than 50%, evidencing their crucial importance in promoting the oxidation of Fe^2+^ into Fe^3+^ ions. The iron content of AjFER and its variants was determined by means of ICP–MS, revealing 67.3 ± 1 atoms per cage for AjFER, 3.1 ± 0.46 atoms per cage for MF protein, 27.2 ± 0.8 atoms per cage for M3 protein, and 3.7 ± 0.4 atoms per cage for M4 protein (Figure S4). After iron uptake, the iron contents of these proteins were determined using ICP–MS, as shown in Figure 6B. The iron contents of the variant ferritins were significantly decreased compared to that of AjFER+Fe^2+^ (*p* < 0.05). The order of iron content among these proteins from high to low was AjFER (2947 ± 54) > M3 (2747 ± 50) > M4 (2064 ± 56) > MF (1474 ± 57) atoms per cage, which was consistent with the color order of the ferritin solutions, which presented from yellow to colorless (AjFER > M3 > M4 > MF) (Figure S2). CD spectroscopy was applied to determine changes in the abundance of secondary structure elements among AjFER and its variants after Fe^2+^ uptake (Figure 7A). The CD spectra of the protein samples containing Fe^2+^ ions indicated a dominant *α*-helix structures with two broad negative minima at 208 and 222 nm (Figure 7A), similar to the above results for AjFER and its variants (Figure 1A). Nevertheless, the percentage content of secondary structure elements for the AjFER, MF, M3, and M4 proteins before and after Fe^2+^ uptake fluctuated, with the exception of the M3 protein (Table S4).

### 3.5. Interaction of Fe^2+^ Ions with AjFER and Its Variants

As a biophysical assay, MST can sensitively measure molecular interactions between target proteins and metal ions in solution [33]. Evidently, the decrease in fluorescence at the heated spot (the MST signal) is altered in the presence of metal ions, thus providing a direct readout of metal ion binding to proteins [38]. To understand why the mutant ferritins lacked the ability to quickly catalyze Fe^2+^ oxidation, we examined the binding affinity of Fe^2+^ ions toward the AjFER, MF, M3, and M4 proteins using MST (Figure 7B). Apart from the MF protein, AjFER and two other mutant proteins presented certain binding affinities for Fe^2+^ ions. The results of the MST binding assays showed that Fe^2+^ ions tightly bound to the proteins in the mid to low nM range, with K_d_ = 94.4 ± 4.2 nM for AjFER. Notably, the M3 and M4 proteins exhibited decreased affinities for Fe^2+^ ions (i.e., weaker binding) and right-shifted the binding curve by approx. two-fold to an apparent K_d_ = 230.6 ± 9.1 nM for the M3 protein and K_d_ = 199.1 ± 8.6 nM for the M4 protein. In contrast, K_d_ = 616.9 ± 14 nM for the MF protein.

### 3.6. Thermal Stability of AjFER and Its Variants

To further study differences in thermostability between AjFER and its variants, we investigated the unfolding temperatures, T_i_, for these protein nanocages using a label-free Tycho measurement, and then confirmed their structural integrity at 90 °C using TEM and DLS analyses (Figure S5). The thermal assays showed that the AjFER, MF, M3, and M4 proteins yielded Ti temperatures of 88.7, 69.8, 87.4, and 74.7 °C, respectively (Figure S5A–D). The TEM micrographs directly observed whether the protein particles were well assembled. As shown in Figure S5E–H, most of the mutant ferritins still exhibited relatively clear shell-like architectures after thermal treatment, compared with AjFER. Nonetheless, the DLS analyses clearly revealed that the predominant D_H_ values of the MF (141.8 ± 8.7 nm), M3 (190.1 ± 7.9 nm), and M4 (122.4 ± 5.4 nm) proteins were greater than that of the AjFER protein (13.5 ± 1.0 nm) in solution (Figure S5I–L), indicating that most of the particles for MF, M3, and M4 were likely aggregates.

## 4. Discussion

Since its discovery, ferritin has been observed to be directly related to the transport and storage of iron in a nontoxic and biologically available form, and it maintains redox balance during iron metabolism for most species of both prokaryotes and eukaryotes [5]. Ferritin has been widely reported in vertebrates and gradually studied in invertebrates. The 3D structures of ferritins for most species exhibit many similarities; however, in contrast to mammalian ferritins, there are some exceptions for ferritins in invertebrate species [7]. For instance, although both Fer147 and PeFer stemmed from the marine invertebrate *Phascolosoma esculenta*, the interiors of their three- and four-fold channels were significantly distinct in terms of amino acid composition and electrostatic potential distribution [13]. In turn, the biochemical characteristics of these channels directly affect their transport properties and determine the preferred transfer pathways of ferrous ions from the exterior of the cage to the ferroxidase center [39]. In previous research, we established a method to express and purify AjFER and solved its crystal structure at a resolution of 2.75 Å [9]. Several critical metal-binding sites were found in AjFER, including six highly conserved residues (Glu25, Tyr32, Glu60, His63, Glu105, and Gln139) at the ferroxidase center, two residues (Asp129 and Glu132) at the three-fold channel, and a Glu168 residue at the four-fold channel. Nonetheless, whether AjFER has a different transport property or iron oxidation function from known ferritins remains to be determined. In this study, we prepared a triple AjFER-E25A/E60A/E105A mutant (MF), a double AjFER-D129A/E132A mutant (M3), and a single AjFER-E168A mutant (M4) by site-directed mutagenesis; heterologously expressed the three mutants; and further performed detailed structural and biochemical characterizations using a multitechnical approach.

To identify the structure of AjFER before and after mutation, several supporting experimental methods were performed, including CD spectrum, DLS, and TEM analyses. The CD spectra revealed that the AjFER, MF, M3, and M4 proteins all had two very similar negative absorption peaks in the far-UV spectrum (Figure 1A), which was in good agreement with the previously reported secondary structures of plant, animal, and bacterial ferritins [13,40,41]. The CD results suggested that such mutations hardly affected the stability and integrity of AjFER, which was fully consistent with the results of the DLS and TEM analyses (Figure 1B–F). To obtain more insight into the structural information, two powerful X-ray techniques (macromolecular X-ray crystallography and SAXS) were used to investigate the 3D structures of the mutant ferritins in crystal and solution states, respectively. Generally, numerous experiments have implied that eight hydrophilic three-fold channels in most ferritins are considered potential gates for the entry of metal ions into the cage with the assistance of highly conserved residues, such as Glu and Asp [12,42]. As previously described [9], the negatively charged residues Asp129 and Glu132 are located at the three-fold pores of AjFER and play a pivotal role in Fe^2+^ translocation to the internal cavity of the protein, at which Fe^2+^ ions reach the ferroxidase center. This is similar to the reported crystal structure of bullfrog M-ferritin (BfMF) [43]. Accordingly, we prepared the M3 protein by site-directed mutagenesis and solved the crystallographic structure of the mutant at a resolution of 1.98 Å (Figure 2). It is well accepted that the internal entrance to the three-fold channel is generally surrounded by regions of the very lowest negative potential at any location in most ferritins, allowing for the attraction and insertion of many divalent ions, such as Fe, Mg, and Mn [12,35]. However, due to replacement with relatively weaker charged alanine residues in the M3 protein, a small group of negative patches located inside the hourglass channel are replaced by neutral electrostatic surface potentials compared to that of AjFER (Figure 3A–C), which most likely affected the entry of metal ions into the cage [39].

Being mostly consistent with the structural features of the D127A/E130A/S131A ferritin variant in BfMF [39], the inherent hour-glass shape of the three-fold channel in the M3 protein was even wider than that in the previous holo-AjFER protein because of substitutions with relatively smaller alanine residues (Figure 3D,E). The diameters of the three three-fold amino acid rings within the three-fold channel of the AjFER and M3 proteins varied significantly, with the inner ring of the D129A residue ranging from ~6.3 to ~9.8 Å, the middle ring of the E132A residue ranging from ~6.0 to ~7.4 Å, and the outer ring of the His116 and Cys128 residues ranging from ~7.5 to ~7.9 Å. Notably, the conformation of the Cys128 residue from the three-fold channel in the M3 protein, corresponding to the Cys126 residue in BfMF [43], was profoundly affected by a single-point mutation (Figure 4A–C). In addition, based on very strong positive electron densities presented in averaged *F*_o_–*F*_c_ maps and the coordination environment of cadmium, three Cd^2+^ ions were observed in the three-fold channel of the M3 protein, and each Cd^2+^ ion was coordinated with the residues His116, Asp120, and Cys128, as well as three water molecules. A metal ion binding to the Cys128 residue (forming the Cd–S–Cys charge transfer complex) at the three-fold channel of ferritin revealed that metal ion coordination to sulfur can complement ionic binding during Fe^2+^ transit through ion channels [43,44]. Additionally, consistent with most ferritins from marine invertebrates [7,9,14,15,45], two metal ion-binding sites (i.e., sites A and B) in the ferroxidase center were observed in the middle of four *α*-helical bundles per subunit in the M3 protein (Figure 4E). On the basis of the positive electron density map and the coordination environment of iron around the ferroxidase site, two iron ions were assigned in sites A and B of the ferroxidase center of the M3 protein. Taking into account the highly conversed residues at the ferroxidase center [9], it can be assumed that both the AjFER and M3 proteins may have some oxidative functions that are similar to those of other marine invertebrates.

Since crystallographic methods may preferentially utilize the most stable conformation of the protein macromolecules and overlook some function-related differences in solution, the structures of AjFER and its variants were investigated using SAXS. The SAXS data revealed that the overall shape and size of the AjFER, MF, M3, and M4 proteins in solution were consistent with the crystal structures of the AjFER [9] and M3 proteins (Figure 2 and Figure 5). Based on calculations using the SAXS data, some structural parameters in solution (e.g., *D_max_*, *R_g_*, and MWs) were similar and consistent with those from the crystal structures (Table S3). In each case, the *R_g_* values, estimated from the Guinier plot and *p(r)* distribution by GNOM, were in agreement with the Guinier analysis, varying between 52.61 and 53.81 Å (Figure 5A–C). The *D_max_* values for AjFER and its variants were distributed from 120 to 126 Å, which was very close to the diameter of the spherically symmetric 24-mer cage with a hollow core [9]. Additionally, the shape of the *p(r)* distribution curves and the Kratky plots indicated that all of the proteins were well folded and had a globular shape (Figure 5A–C). Furthermore, the theoretical scattering curve generated from the AjFER model fit well to the experimental SAXS data (χ^2^ of 52.7 using FoXS) (Figure S3A). However, the results also showed that there were certain differences between the crystal and solution structures of the MF, M3, and M4 proteins (Figure S3B–D). The results implied that the site-directed mutations of the key metal-binding sites had certain effects on the structure of AjFER in solution.

It is well documented that in addition to the iron storage function of AjFER from the sea cucumber (*S. monotuberculatus*), AjFER also plays a dominant role in iron detoxification and the immune response [8]. These unique characteristics of ferritin can not only maintain the cellular and organismic iron homeostasis process but also help to avoid the toxicity of ferrous iron. Similar to most ferritins from other marine invertebrates, the metal-binding sites in AjFER are commonly composed of a ferroxidase center, hydrophilic three-fold channel, and putative four-fold channel [9]. Therefore, we further investigated the catalytic activity of the AjFER, MF, M3, and M4 proteins in converting Fe^2+^ into Fe^3+^ via UV absorption at 315 nm, aiming to elucidate the relationship between iron oxidation activity and the protein–metal binding sites (Figure 6A). It is apparent that the D129A/E132A or E168A substitutions on each subunit of AjFER caused a significant decrease in the rate of Fe^2+^ oxidation by oxygen, in contrast with AjFER. Notably, the amino acid substitutions E25A/E60A/E105A had already seriously affected the catalytic activity for Fe^2+^ ions, possibly resulting in relatively low rates of iron oxidation, which was in agreement with the above results related to the iron contents (Figure 6B). Such results indicated that the substitution of alanine residues for carboxylate ligands of carboxylate residues Asp and Glu most likely influenced the altered ability of the variants to capture and bind metal ions [46]. Further, we determined the secondary structures and binding affinities after interaction between the AjFER, MF, M3, and M4 proteins with Fe^2+^ ions by CD and MST analyses, respectively (Figure 7A,B). The uptake of Fe^2+^ ions within ferritins did not significantly affect the overall structure of these proteins, as revealed by the CD spectra (Figure 7A). The MST results revealed significantly differential binding between AjFER and its variants to Fe^2+^ ions, in which Fe^2+^ had a higher affinity for AjFER (K_d_ value of 94.4 ± 4.2 nM) than for the other variants, and the order of the binding affinity was AjFER > M4 > M3 > MF (Figure 7B). On the basis of these results, it can be roughly concluded that the residues Asp129 and Glu132 at the three-fold channel in AjFER are probably involved in Fe^2+^ capture and its translocation to the internal cavity of the protein, from where Fe^2+^ reaches the ferroxidase center, similar to that proposed for BfMF [43]. Furthermore, our results confirm the ferroxidase center of AjFER indeed has a function in iron oxidation and the Glu168 residue in AjFER can act as a potential metal-ion binding site at the four-fold channel, similar to observations in previous studies [9,14]. In addition, the variants (MF, M3, and M4 proteins) exhibited lower unfolding temperatures than native AjFER, suggesting that mutations of the potential metal-binding sites in AjFER could have varying effects on its thermal stability and structural integrity. However, further research (e.g., using X-ray crystallography combined with site-directed mutagenesis) should provide further evidence regarding the mechanism of Fe^2+^ entry and/or Fe^2+^ binding to the ferroxidase center and the four-fold channel during iron ion transport in AjFER.

## 5. Conclusions

In this study, AjFER and its variants (MF, M3, and M4 proteins) were prepared and their structural and biochemical features were further characterized. The crystal structure of the M3 protein was solved at a resolution of 1.98 Å and the results suggested that D129A/E132A substitutions significantly affected the quaternary structure of the three-fold channel in contrast with that of AjFER. The structural parameters in solution obtained from the SAXS data were mostly in accordance with those stemming from the crystal structure of AjFER. Compared to AjFER, the site-directed mutagenesis of the MF, M3, and M4 proteins seriously affected the thermostability and catalytic activities of these proteins for Fe^2+^ ions, resulting in their relatively low rates of iron oxidation, which was in good agreement with their iron contents and iron-binding affinities. All of these new findings are expected to increase the understanding of iron ion transport in the life-supporting ferritin superfamily.

## Figures and Tables

**Table 1:** X-ray diffraction data and refinement statistics.

Crystal Parameters	AjFER-D129A/E132A Mutant (M3)
**Data collection**	
Beamline	SSRF-BL19U1
Wavelength (Å)	0.97892
Space Group	*C*121
Resolution range (Å)	47.91–1.98 (2.03–1.98) *
Cell dimensions	
a, b, c (Å)	192.83, 130.95, 122.62
*α*, *β*, *γ* (˚)	90.00, 119.40, 90.00
No. of reflections	1238022
Completeness (%)	95.6 (98.4) *
I/σ(I)	2.98 (2.68) *
R*_merge_*	0.085 (0.717) *
Redundancy	6.8 (6.0) *
CC_(1/2)_	0.834 (0.830) *
Wilson B-factor (Å^2^)	25.8
**Refinement**	
R*_work_*/R*_free_*	0.177/0.220
Mean B-values (Å^2^)	28.089
R.m.s. deviations	
Bond lengths (Å)	0.0121
Bond angles (°)	1.4651
Chir volume	0.0778
Ramachandran plot (%)	
Favored regions	97.7
Allowed regions	2.15
Outlier	0.15
PDB ID	7Y74

* Values in parentheses correspond to the highest resolution shell.

## Data Availability

The atomic coordinates and structural factors of AjFER-D129A/E132A mutant have been deposited in the PDB database (http://wwpdb.org/) under the accession code 7Y74.

## Supplementary Materials

The following supporting information can be downloaded at: https://www.mdpi.com/article/10.3390/polym14245378/s1, Figure S1: SDS–PAGE analyses of AjFER and its variants; Figure S2: Solutions of AjFER and its variants after Fe^2+^ uptake; Figure S3: The theoretical scattering curve from the crystal structure of AjFER (PDB code: 7VHR) was fitted into the experimental SAXS data of the (A) AjFER; (B) the AjFER-E25A/E60A/E105A mutant (MF); (C) the AjFER-D129A/E132A mutant (M3); (D) the AjFER-E168A mutant (M4); Figure S4: The determination of the iron contents in the protein samples by ICP–MS; Figure S5: Unfolding profiles of the (A) AjFER, (B) AjFER-E25A/E60A/E105A mutant (MF), (C) AjFER-D129A/E132A mutant (M3) and (D) AjFER-E168A mutant (M4) proteins were measured with Tycho NT.6, yielding inflection temperatures of protein unfolding (T_i_). Transmission electron microscopy (TEM) images of the (E) AjFER, (F) MF, (G) M3 and (H) M4 proteins at 90 °C for 10 min. Scale bars represent 100 nm. (I–L) Dynamic light scattering (DLS) intensity of AjFER and its variants upon thermal treatment at 90 °C for 10 min. Values are represented as the mean ± SD of three replicates; Table S1: The percentage content of secondary structure elements of AjFER and its variants; Table S2: The distances of metal ion coordination in the M3 protein; Table S3: Small-angle X-ray scattering data collection and statistics; Table S4: The percentage content of secondary structure elements of AjFER and its variants after Fe^2+^ uptake.
