# Supplementary material for: Structural and Functional Insights into the Roles of Potential Metal-Binding Sites in Apostichopus japonicus Ferritin
Source: Polymers (Basel). 2022 Dec 8;14(24):5378. doi: 10.3390/polym14245378 (PMC9785301; doi:10.3390/polym14245378)
Supplement: Supplementary file 1 [file polymers-14-05378-s001.zip › polymers-1864976-supplementary.pdf]

polymers

Supplementary Materials

Structural and Functional Insights into the Roles of Potential Metal-Binding Sites in *Apostichopus japonicus* Ferritin

Yan Wu [1,2,3,†], Chunheng Huo [1,3,4,†], Tinghong Ming [1,3,4,*], Yan Liu [5], Chang Su [5], Xiaoting Qiu [2], Chenyang Lu [1,3,4], Jun Zhou [1,3,4], Ye Li [1,3,4], Zhen Zhang [1,3,4], Jiaojiao Han [1,3,4], Ying Feng [1,3,4,6] and Xiurong Su [1,3,4,*]

[1] State Key Laboratory for Managing Biotic and Chemical Threats to the Quality and Safety of Agro-Products, Ningbo University, Ningbo 315211, China
[2] College of Food and Pharmaceutical Sciences, Ningbo University, Ningbo 315832, China
[3] Key Laboratory of Aquacultural Biotechnology Ministry of Education, Ningbo University, Ningbo 315832, China
[4] School of Marine Science, Ningbo University, Ningbo 315832, China
[5] Zhejiang Collaborative Innovation Center for High Value Utilization of Byproducts from Ethylene Project, Ningbo Polytechnic, Ningbo 315800, China
[6] College of Life Sciences, Tonghua Normal University, Tonghua 134000, China
* Correspondence: mingtinghong@nbu.edu.cn (T.M.); suxiurong_public@163.com (X.S.); Tel./Fax: +86-574-87608368 (X.S.)
† These authors contributed equally to this work.

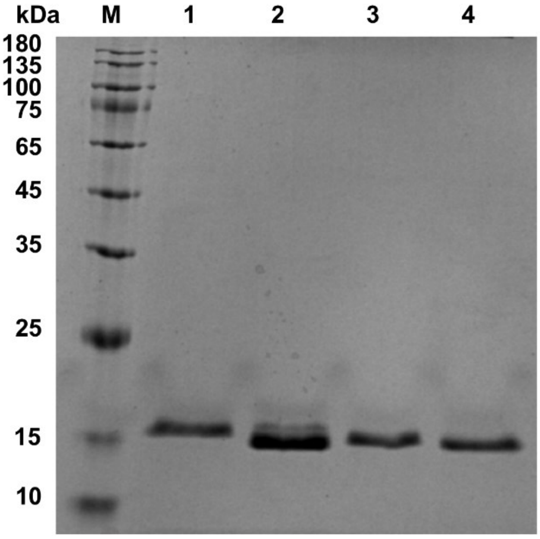

Figure S1. SDS–PAGE analyses of the AjFER and its variants. Lane 1: AjFER, Lane 2: AjFER-E25A/E60A/E105A mutant (MF), Lane 3: AjFER-D129A/E132A mutant (M3), Lane 4: AjFER-E168A mutant (M4).

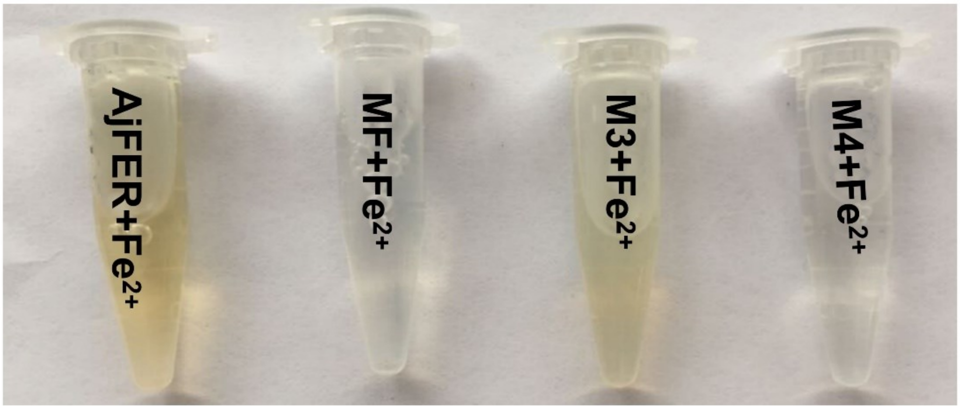

Figure S2. Solutions of AjFER and its variants after Fe^{2+} uptake. AjFER+Fe^{2+}: Fe^{2+}-loaded AjFER, MF+Fe^{2+}: Fe^{2+}-loaded AjFER-E25A/E60A/E105A, M3+Fe^{2+}: Fe^{2+}-loaded AjFER-D129A/E132A, M4+Fe^{2+}: Fe^{2+}-loaded AjFER-E168A.

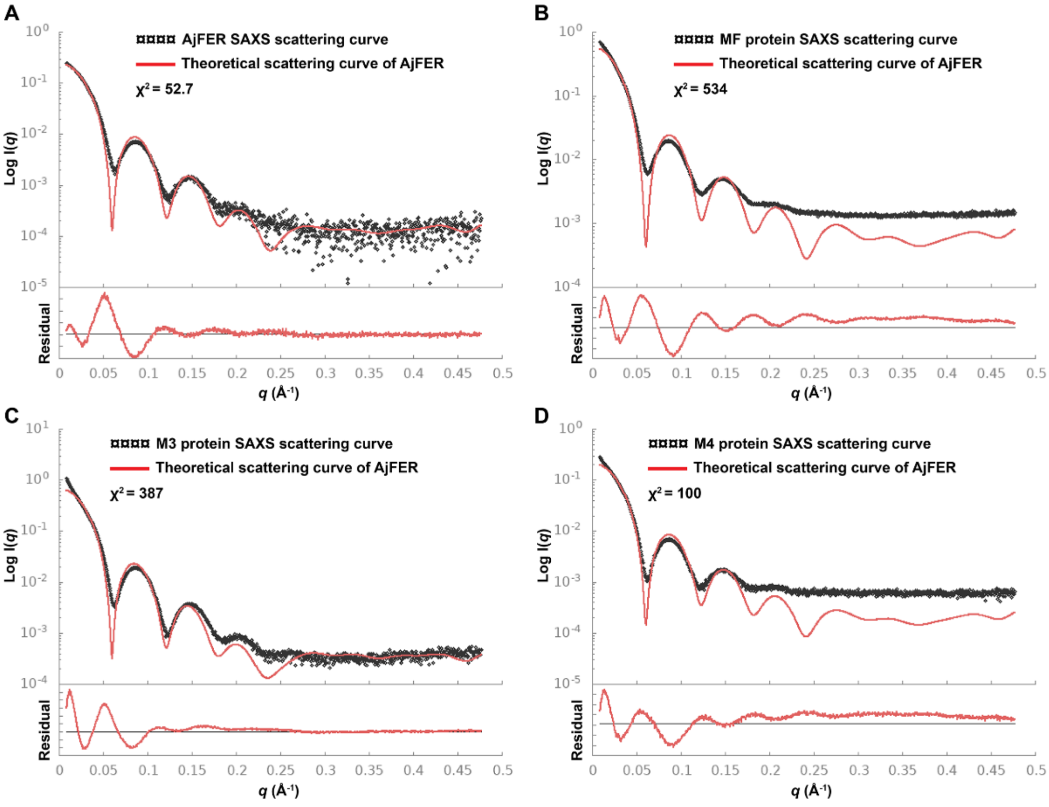

Figure S3. The theoretical scattering curve from the crystal structure of AjFER (PDB code: 7VHR) was fitted into the experimental SAXS data of the (**A**) AjFER; (**B**) the AjFER-E25A/E60A/E105A mutant (MF); (**C**) the AjFER-D129A/E132A mutant (M3); (**D**) the AjFER-E168A mutant (M4).

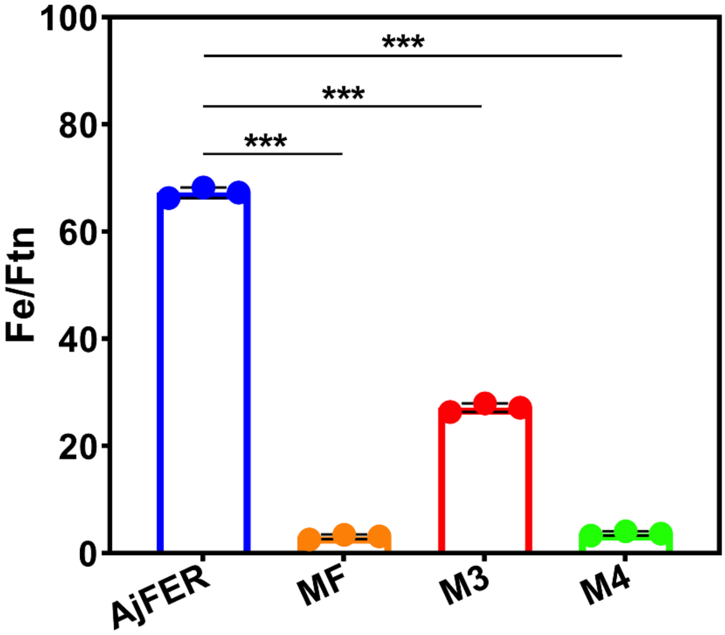

Figure S4. The determination of the iron contents in the protein samples by ICP–MS. MF: AjFER-E25A/E60A/E105A mutant, M3: AjFER-D129A/E132A mutant, M4: AjFER-E168A mutant. ****p*-value <0.001; Control group: AjFER.

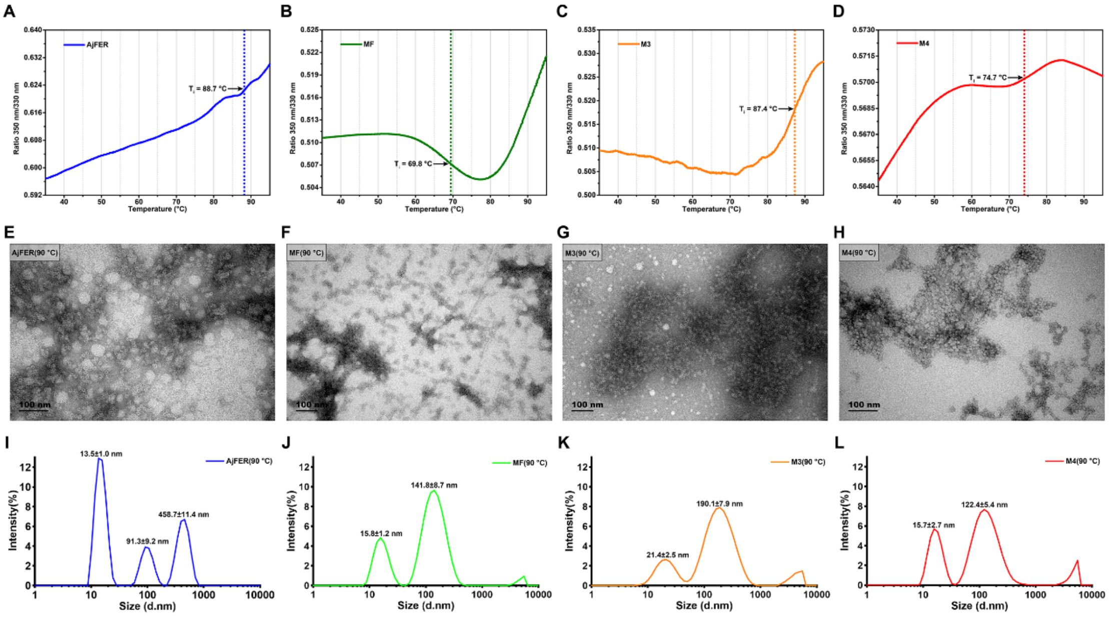

Figure S5. Unfolding profiles of the (**A**) AjFER, (**B**) AjFER-E25A/E60A/E105A mutant (MF), (**C**) AjFER-D129A/E132A mutant (M3) and (**D**) AjFER-E168A mutant (M4) proteins were measured with Tycho NT.6, yielding inflection temperatures of protein unfolding (T_i). Transmission electron microscopy (TEM) images of the (**E**) AjFER, (**F**) MF, (**G**) M3 and (**H**) M4 proteins at 90 °C for 10 min. Scale bars represent 100 nm. (**I**–**L**) Dynamic light scattering (DLS) intensity of the AjFER and its variants upon thermal treatment at 90 °C for 10 min. Values are represented as the mean ± SD of three replicates.

Table S1. The percentage content of secondary structure elements of AjFER and its variants.

	***α*-Helix**	***β*-sheet**	***β*-turn**	**random coil**
AjFER	84.7±1.0	0	15.3±0.6	0
MF	84.9±1.1	3.1±0.6***	9.2±0.6***	2.8±0.5***
M3	82.4±2.4	0	15.7±0.7	1.9±0.4***
M4	86.1±1.0	0	13.9±1.0	0

****p*-value <0.001; Control group: AjFER.

Table S2. The distances of metal ion coordination in the M3 protein.

	**Bond distances (Å)**					
**Fe1**	2.17±0.15 (Glu25)	2.23±0.06 (Glu60)	2.30±0.10 (His63)	2.60±0.10 (Wat1)	-	-
**Fe2**	2.30±0.10 (Glu60)	2.37±0.06 (Glu105)	2.37±0.06 (Wat1)	3.57±0.15 (Wat2)	-	-
**Cd**	2.47±0.21 (Asp120)	2.33±0.06 (His116)	2.37±0.06 (Cys128)	3.70±0.10 (Wat1)	3.53±0.15 (Wat2)	3.50±0.10 (Wat3)

Table S3. Small-angle X-ray scattering data collection and statistics.

	**AjFER**	**MF**	**M3**	**M4**
**Data collection parameters**				
Beamline	SSRF-BL19U2			
Wavelength (Å)	1.24			
q range ($Å^{-1}$)	0.0084-0.4764			
Exposure time (s)	1.0			
Protein concentration (mg/mL)	1~4			
Temperature (K)	293			
**Structural parameters**				
I(0) arbitrary units from Guinier	0.24	0.59	0.66	0.22
I(0) arbitrary units from *p(r)*	0.24	0.59	0.66	0.22
R_g from Guinier (Å)	52.87	53.18	53.58	53.81
R_g from *p(r)* (Å)	52.61	52.94	53.31	53.54
D_{max} (Å)	120	121	125	126
Porod volume estimate ($Å^3$)	606063	731868	674254	712333
MWs from *I*(0) (kDa)	481.3	510.2	882.2	602.8
MWs from sequence (kDa)	480.96	476.88	478.56	479.52
**Software employed**				
Data processing	PRIMUS			
p(r) function calculation	GNOM			
Ab initio modeling	DAMMIF			
Validation and averaging	DAMMIN, DAMMIX			
3-D graphical representation	PyMOL			
**Modeling parameters**				
Discrepancy value (χ^2)	52.7	534	387	100

Table S4. The percentage content of secondary structure elements of AjFER and its variants after Fe^{2+} uptake.

	***α*-Helix**	***β*-sheet**	***β*-turn**	**random coil**
AjFER+Fe^{2+}	82.7±0.6	0	13.1±0.3	4.2±0.2
MF+Fe^{2+}	86.2±0.5***	1.2±0.1***	12.0±0.3**	0.6±0.1***
M3+Fe^{2+}	81.9±0.4	0	15.4±0.4***	2.7±0.1***
M4+Fe^{2+}	82.6±0.5	4.3±0.1***	13.0±0.2	0

***p*-value <0.01, ****p*-value <0.001; Control group: AjFER+Fe^{2+}.
